# Prognostic impact of sleep-disordered breathing on mortality and cardiovascular events in renal dialysis: A meta-analysis

**DOI:** 10.17305/bb.2025.13100

**Published:** 2025-10-10

**Authors:** Tingting Hao, Yingjiao Shen, Xiaofeng Lu

**Affiliations:** 1 Hemodialysis Unit, Shangluo Central Hospital, Shangluo, China; 2 Department of Nephrology, Changzhi People’s Hospital, Changzhi, China; 3 Department of Nephrology, Tongxiang First People’s Hospital, Tongxiang, China

**Keywords:** Sleep-disordered breathing, hemodialysis, peritoneal dialysis, mortality, meta-analysis

## Abstract

Sleep-disordered breathing (SDB) is prevalent among patients undergoing renal dialysis, yet its prognostic implications for mortality and cardiovascular outcomes remain unclear. This meta-analysis investigates the relationship between SDB and all-cause mortality as well as major adverse cardiovascular events (MACEs) within this demographic. A systematic search of PubMed, Embase, and Web of Science was conducted from inception to May 29, 2025, focusing on longitudinal observational studies that assessed SDB in adult dialysis patients. The primary outcome analyzed was all-cause mortality, while the secondary outcome was MACEs. Pooled hazard ratios (HRs) with 95% confidence intervals (CIs) were computed using random-effects models to account for heterogeneity. A total of eleven cohort studies encompassing 656,328 dialysis patients, of which 23,725 had SDB, were included. The results indicated that SDB was significantly associated with an increased risk of all-cause mortality (HR: 1.79, 95% CI: 1.42–2.25; *I*^2^ ═ 32%; *P* < 0.001). Notably, the association was more pronounced in Asian studies (HR: 2.07) compared to non-Asian studies (HR: 1.35; *P* for subgroup difference = 0.008) and in studies employing polysomnography or pulse oximetry vs those using ICD codes (HR: 2.57 and 2.00 vs 1.35; *P* ═ 0.002). Furthermore, five studies indicated that SDB was linked to an elevated risk of MACEs (HR: 2.68, 95% CI: 1.86–3.85; *I*^2^ ═ 0%; *P* < 0.001). In conclusion, SDB is associated with heightened mortality and cardiovascular risk in patients on renal dialysis. These findings underscore the necessity for increased awareness and management of SDB in this population. However, further interventional studies are required to ascertain whether systematic screening and treatment can enhance clinical outcomes.

## Introduction

End-stage renal disease (ESRD) represents a significant global health challenge, with its increasing incidence attributed to population aging and the rising prevalence of diabetes, hypertension, and other chronic kidney diseases [1, 2]. Renal replacement therapy, primarily through dialysis or kidney transplantation, is crucial for maintaining the lives of patients with ESRD [3]. Dialysis methods, including hemodialysis (HD) and peritoneal dialysis (PD), effectively eliminate uremic toxins, excess fluid, and correct electrolyte imbalances, thereby alleviating symptoms associated with renal failure [4]. Nevertheless, patients undergoing dialysis experience significantly reduced survival rates compared to the general population, primarily due to complications such as cardiovascular disease and infections [5, 6]. Therefore, identifying novel, modifiable predictors of poor prognosis in dialysis patients is essential for facilitating earlier interventions and improving clinical outcomes [7].

Sleep-disordered breathing (SDB) represents a range of conditions characterized by abnormal respiration during sleep, including obstructive sleep apnea, central sleep apnea, and mixed apnea [8, 9]. Diagnosis typically employs objective methods such as polysomnography, portable sleep monitoring, or overnight pulse oximetry, utilizing indices like the apnea–hypopnea index (AHI) or oxygen desaturation index (ODI) to define severity [10, 11]. Beyond inducing sleep fragmentation and hypoxemia, SDB is associated with hypertension, obesity, diabetes, and dyslipidemia—all established risk factors for cardiovascular morbidity and mortality [8, 9]. These associations underscore the potential for SDB to exacerbate vascular and metabolic burdens in susceptible populations [8, 9]. Recent studies have also highlighted the systemic vascular implications of SDB, including alterations in retinal microvasculature [12], further corroborating its extensive impact on cardiovascular health. In dialysis patients, SDB may contribute to adverse outcomes through mechanisms such as intermittent hypoxia, sympathetic overactivity, endothelial dysfunction, inflammation, and metabolic disturbances, which can worsen cardiovascular disease and accelerate mortality [13, 14]. Although SDB has been extensively investigated in the general population and in non-dialysis chronic kidney disease, evidence regarding its prevalence and impact in dialysis populations remains limited and inconsistent, with studies varying widely in design, sample size, diagnostic criteria, and adjustment for confounding variables [15–25]. To address these discrepancies and provide a clearer estimate of the prognostic implications of SDB, we conducted a meta-analysis of longitudinal observational studies to evaluate the relationship between SDB and all-cause mortality in adult patients undergoing renal dialysis, with major adverse cardiovascular events (MACEs) as a secondary outcome.

## Materials and methods

This study adhered to the PRISMA 2020 guidelines [26] and the Cochrane Handbook for Systematic Reviews of Interventions [27], ensuring methodological rigor in the selection of studies, data extraction, statistical analysis, and interpretation of results. The protocol was prospectively registered with PROSPERO (ID: CRD420251121897).

### Literature search

A comprehensive literature search was conducted using PubMed, Embase, and Web of Science, employing an extensive set of search terms that incorporated the following keywords and concepts: (1) “sleep disordered breathing” OR “sleep breathing disorders” OR “sleep apnea syndrome” OR “obstructive sleep apnea” OR “obstructive sleep apnea syndrome” OR “obstructive sleep hypopnea syndrome” OR “OSAHS” OR “OSAS” OR “sleep apnea”; (2) “dialysis” OR “hemodialysis” OR “peritoneal dialysis”; and (3) “mortality” OR “death” OR “deaths” OR “prognosis” OR “survival” OR “adverse events” OR “cardiovascular”. The search was restricted to human studies and included only full-text articles published in English in peer-reviewed journals. To ensure comprehensiveness, we also manually screened the reference lists of relevant original and review articles for additional eligible studies. The search encompassed all publications from database inception until May 29, 2025. The detailed search strategy for each database is provided in the Supplemental File 1.

### Study eligible criteria

We utilized the PICOS framework to define the inclusion criteria. Population (P): Adult patients (≥18 years) undergoing renal dialysis, including HD or PD, regardless of dialysis vintage, sex, or comorbidities.

Intervention/Exposure (I): Presence of SDB, including obstructive sleep apnea, central sleep apnea, or mixed apnea, confirmed through objective diagnostic methods consistent with those used in the original studies (e.g., polysomnography, home sleep apnea testing, or clinical diagnostic criteria). Additionally, validated hypoxemia indices such as the ODI or average SaO_2_ were accepted as objective surrogates for SDB in dialysis populations.

Comparison (C): Patients on renal dialysis without SDB.

Outcomes (O): The primary outcome is all-cause mortality, and the secondary outcome is the composite outcome of MACEs, which generally include myocardial infarction, heart failure, stroke, and cardiovascular deaths, compared between patients with and without SDB.

Study design (S): Observational studies with longitudinal follow-up (prospective or retrospective cohort studies) that report risk estimates (e.g., hazard ratios (HRs), relative risks, or odds ratios) for the association between SDB and the outcomes of interest.

Studies were excluded if they: (1) did not involve renal dialysis patients; (2) diagnosed SDB solely based on patient-reported symptoms or questionnaires; (3) lacked a comparator group without SDB or failed to stratify patients based on SDB status; (4) were cross-sectional, case reports, editorials, reviews, or conference abstracts without full-text data; (5) did not report all-cause mortality or MACEs as outcomes; (6) provided insufficient data to extract or calculate effect estimates with 95% confidence intervals (CIs); or (7) were duplicate publications using the same cohort data without additional relevant information. In cases of overlapping populations, only the study with the largest sample size was retained for inclusion in the meta-analysis.

### Study quality evaluation

Two reviewers independently conducted the literature search, screened studies, assessed methodological quality, and extracted data. Discrepancies were resolved through consultation with the corresponding author. The quality of included studies was evaluated using the Newcastle–Ottawa Scale (NOS) [28], which examines study selection, control of confounding variables, and outcome assessment. The NOS assigns scores ranging from 1 to 9, with a score of 7 or above indicating high methodological quality.

### Data collection

Data collected for the meta-analysis included study details (author, year, country, and design), patient characteristics (sample size, mean age, sex distribution, and type of dialysis received), exposure details (diagnostic methods for SDB and the number of patients with SDB at baseline), mean follow-up durations, outcomes reported, numbers of patients who died or developed MACEs during follow-up, and covariates adjusted for in the regression models.

**Figure 1.**
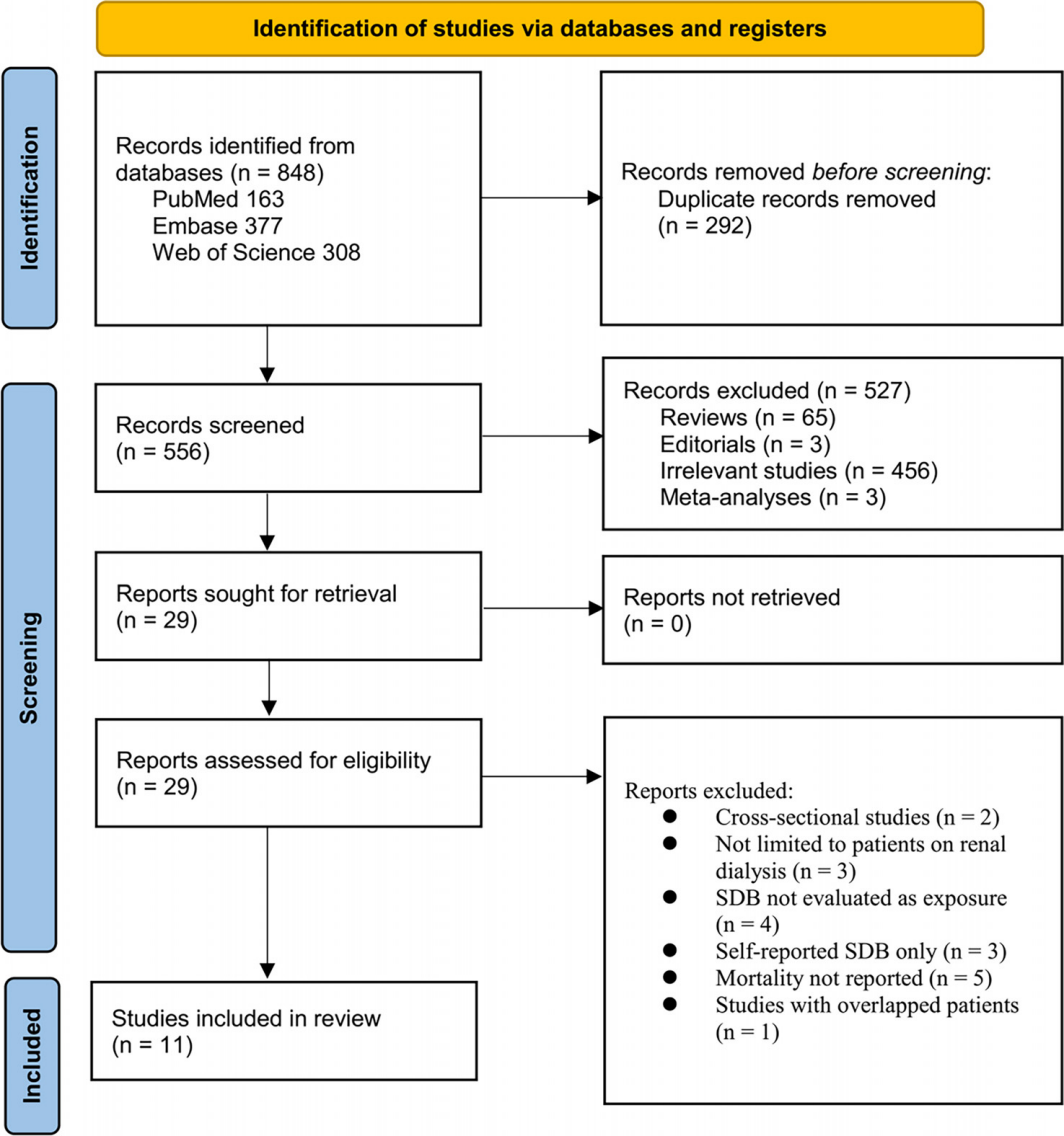
Flowchart of database search and study inclusion.

### Statistical analysis

We utilized HRs and 95% CIs to assess the association between SDB and clinical outcomes in patients on renal dialysis. HRs and their standard errors were either directly extracted or derived from reported 95% CIs or *P* values, followed by logarithmic transformation to stabilize variance and achieve a normal distribution [27]. If multiple HRs were reported from different models, we selected the one with the most comprehensive adjustment. When HRs and 95% CIs were not directly reported, we reconstructed them from published Kaplan–Meier curves using the Parmar/Tierney approach [29]. Heterogeneity was evaluated using the Cochrane *Q* test and the *I*^2^ statistic [30], with a *P* value < 0.10 indicating significant heterogeneity and *I*^2^ values of < 25%, 25%–75%, and > 75% denoting low, moderate, and high heterogeneity, respectively. Random-effects models were applied using the DerSimonian–Laird inverse-variance method in RevMan, which provides pooled estimates incorporating between-study variance (τ^2^) [27]. In addition to the conventional 95% CI, we calculated 95% prediction intervals (PI), which estimate the expected range of effects in future studies [27]. Alongside the DerSimonian–Laird random-effects model, we conducted sensitivity analyses using the Hartung–Knapp–Sidik–Jonkman (HKSJ) method to generate more robust CIs, particularly given the moderate number of studies included [27]. To assess the stability of the results, sensitivity analyses were performed by sequentially excluding each study. For the primary outcome of all-cause mortality, predefined subgroup analyses were conducted based on study country (Asian vs non-Asian), design (prospective vs retrospective), type of renal dialysis (HD vs PD), diagnostic methods for SDB, follow-up durations, and study quality scores. Subgroup analyses were stratified using the median values of continuous variables to ensure balanced groupings. Publication bias was evaluated through funnel plot visualization and assessed for asymmetry using Egger’s regression test [31]. To further assess small-study effects, we applied the trim-and-fill method, which estimates the number of potentially missing studies and recalculates the pooled effect after imputing them [31]. All analyses were performed using RevMan (version 5.1; Cochrane Collaboration, Oxford, UK) and Stata (version 12.0; Stata Corporation, College Station, TX, USA).

## Results

### Study inclusion

The study selection process is illustrated in Figure 1. Initially, 848 records were identified from three databases. After removing 292 duplicates, 556 articles were screened based on their titles and abstracts. Of these, 527 were excluded for not meeting the meta-analysis objectives. The remaining 29 full-text articles were independently evaluated by two reviewers, leading to the exclusion of 18 studies for specific reasons outlined in Figure 1. Ultimately, 11 studies were included in the analysis [15–25].

**Table 1:** Characteristics of the included studies

**Study**	**Country**	**Design**	**No. of patients**	**Mean age (years)**	**Men (%)**	**Dialysis type**	**Diagnosis of SDB**	**No. of patients with SDB**	**Follow-up duration (months)**	**Outcomes reported**	**No. of patients died**	**No. of patients with MACEs**	**Variables adjusted**
Zoccali, 2002	Italy	PC	50	50.1	62	Mixed (HD: 40; PD: 10)	Nocturnal pulse oximetry (average SaO_2_ <95%)	9	32	Mortality and MACEs	13	19	Age, cholesterol, LVMI, BP, smoking, dialysis vintage
Jung, 2010	Korea	PC	30	55.8	70	HD	PSG (Sleep time SaO_2_ < 90%)	25	48	Mortality	14	NA	Age
Tang, 2010	Hong Kong, China	PC	93	55.3	51.6	PD	PSG (AHI ≥ 15)	51	41	Mortality and MACEs	30	53	Age, sex, diabetes, dialysis vintage, residual renal function, minimal nocturnal SaO_2_
Masuda, 2011	Japan	PC	94	64.4	53.2	HD	Pulse oximetry (3% ODI ≥5 events/hour)	44	55	Mortality and MACEs	25	40	Age, sex, diabetes, serum albumin, cardiothoracic ratio
Sivalingam, 2013	UK	PC	91	60.2	66	HD	Limited sleep study (PSG AHI ≥15 + ODI ≥15 or ESS >10)	40	44	Mortality	25	NA	Age, BMI, CRP, cancer status
Kerns, 2018	USA	PC	558	56	56	HD	Clinically diagnosed SDB according to medical records involving PSG	66	23.2	Mortality	104	NA	Age, sex, ethnicity, BMI, CCI, atrial fibrillation, left ventricular mass index, and average intradialytic weight change
Huang, 2018	Taiwan, China	RC	9987	53.7	46.1	PD	ICD-9 codes sleep study confirmation for OSA	70	44.6	Mortality	NR	NA	Age, sex, CAD, diabetes, stroke, hyperlipidemia, COPD, hypertension, CHF, obesity
Harmon, 2018	Brazil	PC	55	50.9	49	HD	PSG (AHI ≥ 5)	40	45	Mortality and MACEs	9	9	None
Kang, 2021	Korea	PC	103	56	67	PD	PSG (AHI ≥ 15)	57	70	Mortality	19	NA	Age, sex, BMI, diabetes, CVD, neck/abdominal circumference, fat tissue index, ECW, hemoglobin, serum albumin
Mochida, 2023	Japan	RC	134	67	64.2	HD	Overnight pulse oximetry (3% ODI)	12	37	Mortality and MACEs	60	71	Age, sex, BMI, HD duration, diabetes, CRP
Prabu, 2023	USA	RC	645133	54.3	56.4	HD	ICD-9 codes + sleep study confirmation for OSA	23311	70	Mortality	421474	NA	Age, sex, race, ethnicity, access type, ESRD etiology, tobacco/alcohol use, hypertension, diabetes, heart failure, arrhythmias, CVD

Note: Event counts represent first events per patient, where applicable, as reported in the original studies. For Huang 2018, only the peritoneal dialysis (PD) subgroup was used in the analysis. Abbreviations: PC: Prospective cohort; RC: Retrospective cohort; HD: Hemodialysis; PD: Peritoneal dialysis; PSG: Polysomnography; SaO_2_: Arterial oxygen saturation; AHI: Apnea-hypopnea index; ODI: Oxygen desaturation index; ESS: Epworth sleepiness scale; MACEs: Major adverse cardiovascular events; LVMI: Left ventricular mass index; BP: Blood pressure; BMI: Body mass index; CRP: C-reactive protein; CCI: Charlson comorbidity index; CAD: Coronary artery disease; COPD: Chronic obstructive pulmonary disease; CHF: Congestive heart failure; CVD: Cardiovascular disease; ECW: Extracellular water; ESRD: End-stage renal disease; NR: Not reported; NA: Not applicable; ICD-9: International classification of diseases, ninth revision.

**Table 2:** Study quality evaluation via the Newcastle–Ottawa scale

**Cohort study**	**Representativeness of the exposed cohort**	**Selection of the non-exposed cohort**	**Ascertainment of exposure**	**Outcome not present at baseline**	**Control for age**	**Control for other confounding factors**	**Assessment of outcome**	**Enough long follow-up duration**	**Adequacy of follow-up of cohorts**	**Total**
Zoccali, 2002	1	1	0	1	1	1	1	1	1	8
Jung, 2010	1	1	0	1	1	0	1	1	1	7
Tang, 2010	1	1	1	1	1	1	1	1	1	9
Masuda, 2011	1	1	0	1	1	1	1	1	1	8
Sivalingam, 2013	1	1	0	1	1	1	1	1	1	8
Kerns, 2018	1	1	0	1	1	1	1	0	1	7
Huang, 2018	0	1	1	1	1	1	1	1	1	8
Harmon, 2018	1	1	1	1	0	0	1	1	1	7
Kang, 2021	1	1	1	1	1	1	1	1	1	9
Mochida, 2023	0	1	1	1	1	1	1	1	1	8
Prabu, 2023	0	1	0	1	1	1	1	1	1	7

Note: NOS domains were judged as follows. Representativeness of the exposed cohort: prospective, consecutive, or random enrollment (all studies recruited consecutive or incident dialysis patients). Selection of the non-exposed cohort: contemporaneous dialysis patients without SDB drawn from the same base population. Ascertainment of exposure: all studies used objective diagnostic methods (polysomnography, overnight oximetry, or validated ICD codes confirmed by sleep studies), yes if diagnostic criteria clearly stated. Outcome not present at baseline: studies excluded patients with prior cardiovascular outcomes when analyzing incident eventsv Control for age: all multivariable models included age. Control for other confounding factors: most studies additionally adjusted for sex, diabetes, BMI, cardiovascular comorbidity, or dialysis vintage. Assessment of outcome: outcomes were obtained from adjudicated medical records, registry linkage, or standardized criteria. Sufficient follow-up duration: all had ≥24 months of median/mean follow-up (range ∼32–70 months). Adequacy of follow-up of cohorts: ≥90% complete follow-up was achieved in each study.

### Summary of study characteristics

The characteristics of the 11 studies included in this meta-analysis are summarized in Table 1. Collectively, these studies encompassed 656,328 adult patients undergoing renal dialysis. They were conducted in various geographic regions, including Italy, Korea, Hong Kong (China), Japan, the United Kingdom, the United States, Taiwan (China), and Brazil, and were published between 2002 and 2023. Eight studies employed prospective cohort designs [15–20, 22, 23], while three were retrospective [21, 24, 25]. The mean age of participants ranged from 50.1 to 67.0 years, with the proportion of male patients varying from 46.1% to 70.0%. Dialysis modalities included HD [16, 18–20, 22, 24, 25], PD [17, 21, 23], or both [15], with the majority focusing on HD (*n* ═ 7). Diagnosis of sleep apnea was based on objective methods, including polysomnography [16, 17, 19, 20, 22, 23], overnight pulse oximetry [15, 18, 24], or through International Classification of Disease (ICD) codes accompanied by sleep study confirmation [21, 25], with diagnostic criteria varying across studies. Consequently, a total of 23,725 patients were identified with SDB at baseline. Follow-up durations ranged from 23.2 to 70.0 months. All studies reported the primary outcome of all-cause mortality [15–25], while five studies also evaluated MACEs [15, 17, 18, 20, 24]. MACEs were defined across studies as composite cardiovascular outcomes, typically including cardiovascular death, myocardial infarction, stroke or transient ischemic attack, heart failure, arrhythmia, peripheral artery disease, and other significant thrombotic or revascularization events, as detailed in Table S1. Most studies [15–19, 21–25] adjusted for important confounders such as age, sex, comorbidities, and dialysis-related factors to varying extents, while one study [20] reported only univariate results.

### Study quality

Study quality was evaluated using the NOS, with scores ranging from 7 to 9, indicating moderate to high methodological quality (Table 2). Two studies achieved the maximum score of 9, reflecting strong representativeness, robust exposure and outcome ascertainment, and adequate control for confounding factors [17, 23]. Five studies scored 8 [15, 18, 19, 21, 24], primarily due to limited exposure ascertainment or representativeness of the exposed cohort. The remaining four studies scored 7 [16, 20, 22, 25], mainly due to incomplete adjustment for confounders or less rigorous exposure assessment. Overall, the included studies demonstrated adequate follow-up durations and reliable outcome measurements, bolstering the robustness of the pooled estimates in this meta-analysis.

**Figure 2.**
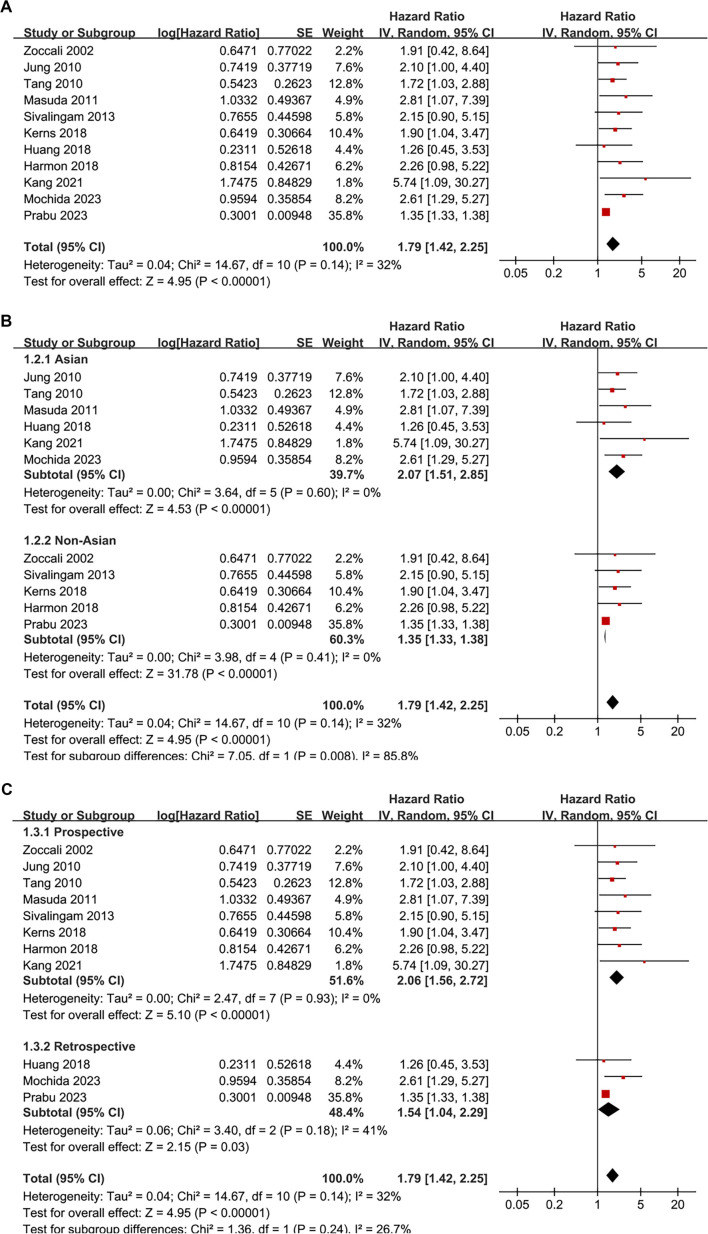
**Association between SDB and all-cause mortality in patients on renal dialysis.** (A) Forest plot of the pooled HR for all-cause mortality in 11 cohort studies; (B) Subgroup analysis stratified by geographic region (Asian vs non-Asian populations); (C) Subgroup analysis stratified by study design (prospective vs retrospective studies). The pooled random-effects model demonstrated that SDB was associated with a significantly increased risk of all-cause mortality (HR: 1.79, 95% CI: 1.42–2.25, *P* < 0.001), with moderate heterogeneity (*I*^2^ ═ 32%). Abbreviations: SDB: Sleep-disordered breathing; HR: Hazard ratio; CI: Confidence interval; SE: Standard error; IV: Inverse variance; df: Degrees of freedom.

### Association between SDB and all-cause mortality

A total of 11 cohort studies [15–25], including one study reporting HRs and 95% CIs derived from Kaplan–Meier curves [21], examined the association between SDB and all-cause mortality in patients undergoing renal dialysis. Moderate heterogeneity was observed (*P* for the Cochrane *Q* test = 0.14; *I*^2^ ═ 32%; τ^2^ ═ 0.04). Pooled results from a random-effects model indicated that SDB was associated with an increased risk of all-cause mortality in these patients (HR: 1.79, 95% CI: 1.42–2.25, *P* < 0.001; Figure 2A). The 95% PI ranged from 1.06 to 3.02, suggesting that most future studies are likely to demonstrate an adverse association. Further meta-analysis utilizing the HKSJ method yielded consistent results (HR: 1.79, 95% CI: 1.32–2.44; Figure S1A), further affirming the robustness of the association.

Sensitivity analyses conducted by sequentially excluding individual datasets confirmed the stability of the results (HR: 1.64–2.06, *P* < 0.05 for all comparisons; Table 3). Specifically, a sensitivity analysis confined to studies employing multivariate analyses that adjusted for age [15–19, 21–25] also yielded consistent findings (HR: 1.76, 95% CI: 1.39–2.23, *P* < 0.001; *I*^2^ ═ 32%). Additionally, excluding the study by Prabu et al. [25] resulted in similar findings while significantly reducing heterogeneity (HR: 2.06, 95% CI: 1.60–2.65, *P* < 0.001; *I*^2^ ═ 0%).

**Table 3:** Sensitivity analyses results by excluding one study at a time

**Mortality**
**Study excluded**	**HR (95% CI)**	***I* ^2^**	***P* for Cochrane *Q* test**	***P* for effect**
Zoccali, 2002	1.81 [1.42, 2.31]	38%	0.11	<0.001
Jung, 2010	1.77 [1.39, 2.25]	32%	0.15	<0.001
Tang, 2010	1.85 [1.42, 2.41]	35%	0.13	<0.001
Masuda, 2011	1.71 [1.37, 2.14]	28%	0.19	<0.001
Sivalingam, 2013	1.78 [1.40, 2.26]	34%	0.14	<0.001
Kerns, 2018	1.80 [1.40, 2.31]	33%	0.13	<0.001
Huang, 2018	1.86 [1.44, 2.38]	39%	0.10	<0.001
Harmon, 2018	1.76 [1.39, 2.23]	32%	0.15	<0.001
Kang, 2021	1.68 [1.37, 2.06]	24%	0.23	<0.001
Mochida, 2023	1.64 [1.34, 2.02]	20%	0.25	<0.001
Prabu, 2023	2.06 [1.60, 2.65]	0%	0.93	<0.001
**MACEs**				
**Study excluded**	**HR (95% CI)**	***I* ^2^**	***P* for Cochrane *Q* test**	***P* for effect**
Zoccali, 2002	2.49 [1.70, 3.66]	0%	0.74	<0.001
Tang, 2010	2.65 [1.67, 4.20]	0%	0.47	<0.001
Masuda, 2011	2.58 [1.72, 3.87]	0%	0.49	<0.001
Harmon, 2018	2.81 [1.93, 4.09]	0%	0.67	<0.001
Mochida, 2023	2.88 [1.88, 4.40]	0%	0.55	<0.001

Abbreviations: HR: Hazard ratio; CI: Confidence interval.

**Figure 3.**
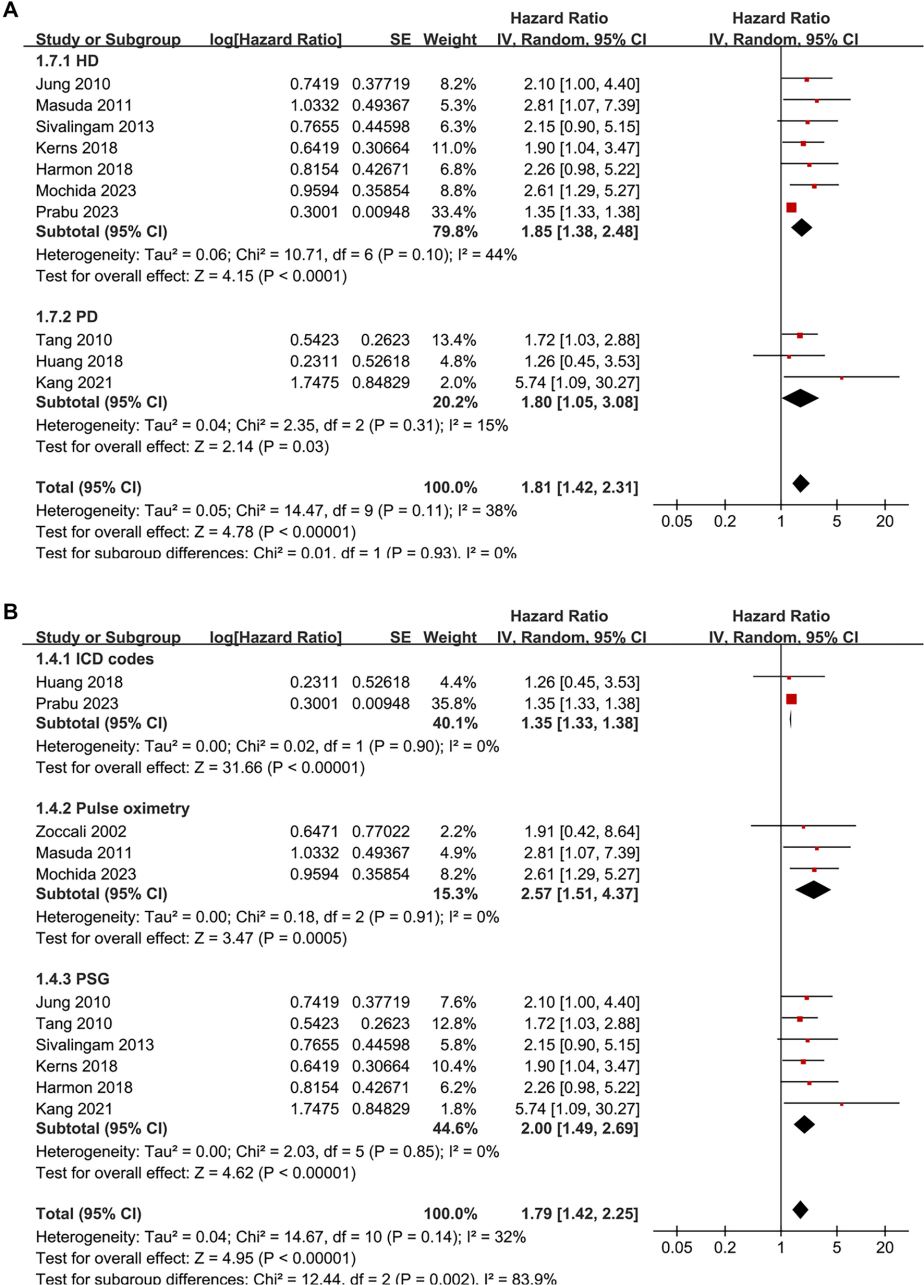
**Subgroup analyses of the association between SDB and all-cause mortality in patients on renal dialysis.** (A) Subgroup analysis stratified by dialysis modality (HD vs PD) showed no significant difference between groups (*P* for subgroup difference = 0.93); (B) Subgroup analysis stratified by diagnostic method demonstrated a stronger association when SDB was diagnosed using overnight pulse oximetry or PSG, compared to ICD codes (*P* for subgroup difference = 0.002). Abbreviations: SDB: Sleep-disordered breathing; HD: Hemodialysis; PD: Peritoneal dialysis; HR: Hazard ratio; CI: Confidence interval; SE: Standard error; IV: Inverse variance; df: Degrees of freedom; ICD: International classification of diseases; PSG: Polysomnography.

**Figure 4.**
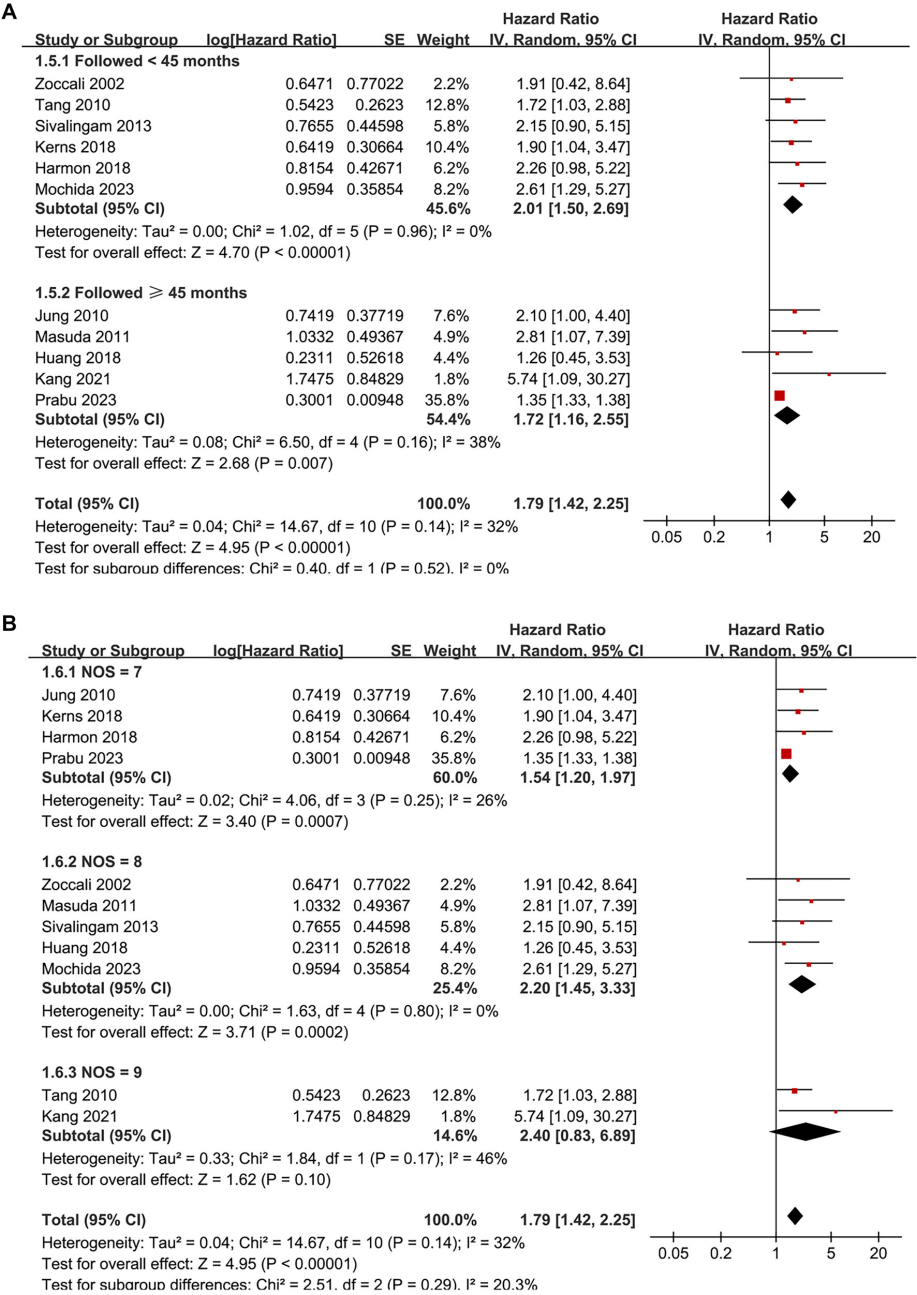
**Subgroup analyses of the association between SDB and all-cause mortality in patients on renal dialysis.** (A) Subgroup analysis stratified by follow-up duration (< 45 months vs ≥ 45 months) showed consistent results, with no significant difference between groups (*P* for subgroup difference = 0.52); (B) Subgroup analysis stratified by study quality scores also showed similar results, without significant subgroup differences (*P* for subgroup difference = 0.29). Abbreviations: SDB: Sleep-disordered breathing; HR: Hazard ratio; CI: Confidence interval; SE: Standard error; IV: Inverse variance; df: Degrees of freedom; NOS: Newcastle–Ottawa scale.

Subsequent subgroup analyses indicated a stronger association between SDB and mortality in dialysis patients from Asian countries compared to their non-Asian counterparts (HR: 2.07 vs 1.35, *P* for subgroup difference = 0.008; Figure 2B). No significant differences were observed between prospective and retrospective studies (*P* for subgroup difference = 0.24; Figure 2C), or among studies including patients on HD or PD (*P* for subgroup difference = 0.93; Figure 3A). A more pronounced association between SDB and mortality was noted in studies diagnosing SDB through overnight pulse oximetry or polysomnography compared to those using ICD codes (HR: 2.57 and 2.00 vs 1.35, *P* for subgroup difference = 0.002; Figure 3B). Finally, similar results were observed between studies with follow-up durations of less than or equal to 45 months compared to those with longer follow-ups (*P* for subgroup difference = 0.52; Figure 4A), and across studies with varying quality scores (*P* for subgroup difference = 0.29; Figure 4B).

### Association between SDB and MACEs

**Figure 5.**
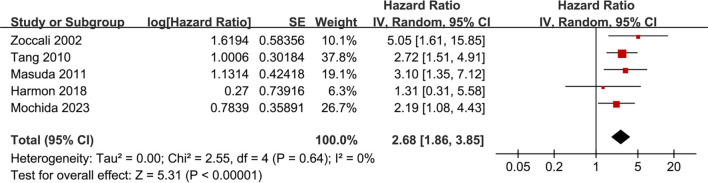
**Association between SDB and MACEs in patients on renal dialysis.** Meta-analysis of five studies demonstrated that SDB was associated with a significantly higher risk of MACEs (HR: 2.68, 95% CI: 1.86–3.85, *P* < 0.001), with no significant heterogeneity (*I*^2^ ═ 0%, *P* ═ 0.64). Abbreviations: SDB: Sleep-disordered breathing; MACEs: Major adverse cardiovascular events; HR: Hazard ratio; CI: Confidence interval; SE: Standard error; IV: Inverse variance; df: Degrees of freedom.

Further meta-analysis of five studies [15, 17, 18, 20, 24] demonstrated that SDB was associated with an elevated risk of MACEs in these patients (HR: 2.68, 95% CI: 1.86–3.85, *P* < 0.001; Figure 5), with no significant heterogeneity observed (*P* for the Cochrane *Q* test = 0.64; *I*^2^ ═ 0%; τ^2^ ═ 0). The 95% PI was 1.48–4.84, closely overlapping with the CI and reinforcing the robustness of the findings. Similarly, further meta-analysis utilizing the HKSJ method confirmed the association between SDB and MACEs (HR: 2.68, 95% CI: 1.77–4.06; Figure S1B). Sensitivity analyses excluding individual studies did not materially alter the results (HR: 2.49–2.88, *P* all < 0.05; Table 3). Specifically, a sensitivity analysis limited to studies employing multivariate analyses [15, 17, 18, 24] also produced consistent results (HR: 2.81, 95% CI: 1.93–4.09, *P* < 0.001; *I*^2^ ═ 0%).

### Publication bias

**Figure 6.**
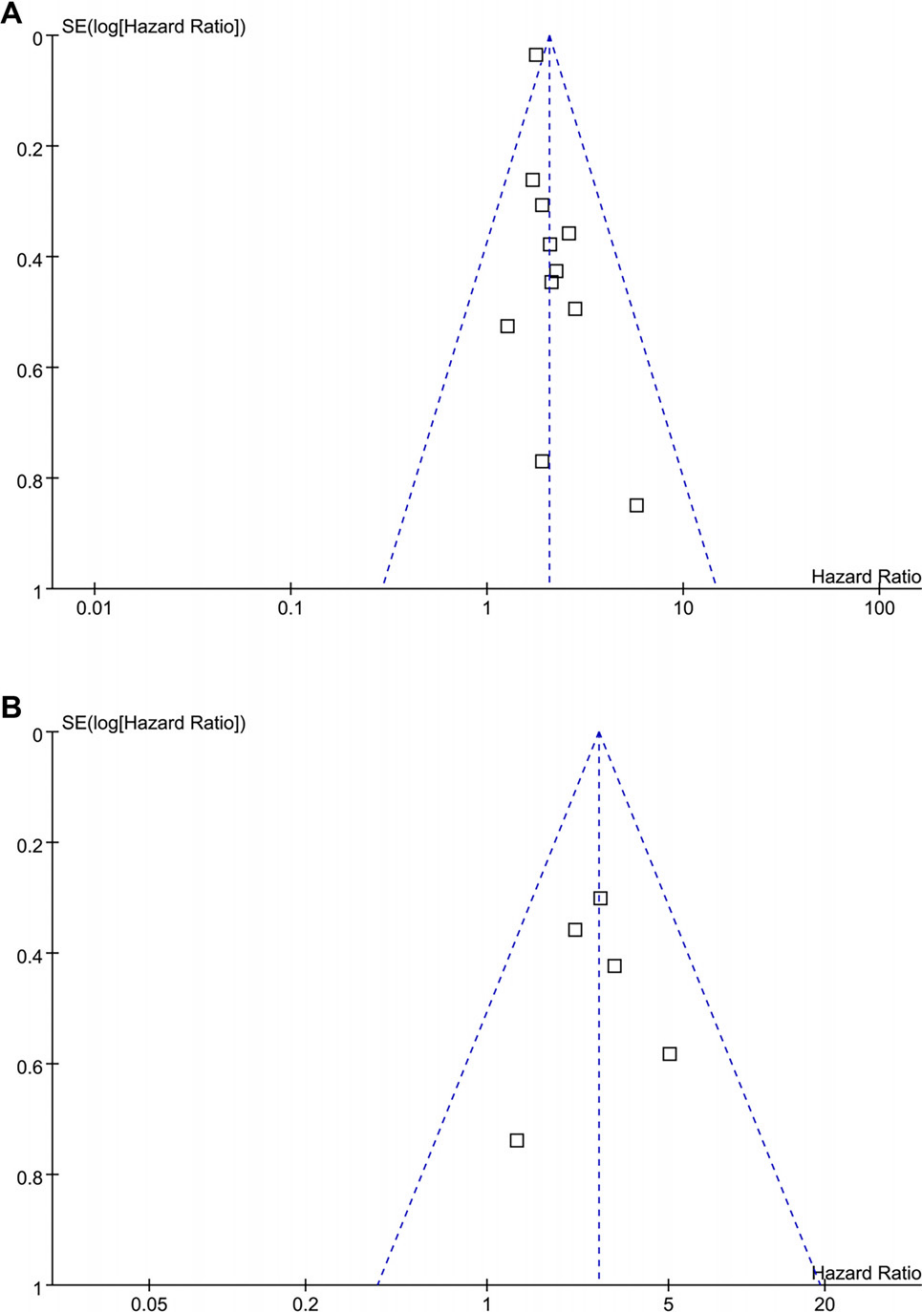
**Funnel plots for estimating the potential publication biases underlying the meta-analyses of the association between SDB and clinical outcomes of patients on renal dialysis.** (A) Funnel plots for the meta-analysis of the association between SDB and all-cause mortality; (B) Funnel plots for the meta-analysis of the association between SDB and MACEs. Abbreviations: SDB: Sleep-disordered breathing; MACEs: Major adverse cardiovascular events.

Funnel plots evaluating the association between SDB and clinical outcomes in dialysis patients are presented in Figure 6A and 6B. For mortality (*k* ═ 11), visual inspection of the funnel plot suggested approximate symmetry, although interpretation is limited by the small number of studies (Figure 6A). Egger’s test did not indicate significant asymmetry (intercept = 0.42, *P* ═ 0.44). A trim-and-fill analysis did not impute any additional studies, and a selection model yielded results consistent with the primary analysis, suggesting that the observed association was not influenced by small-study effects. For MACEs (*k* ═ 5), the funnel plot was not formally tested due to insufficient power; publication bias was assessed descriptively (Figure 6B).

## Discussion

This meta-analysis provides robust evidence that SDB is associated with an increased risk of all-cause mortality and MACEs in patients undergoing renal dialysis. Sensitivity analyses restricted to multivariable-adjusted studies demonstrated a consistent association, suggesting that the observed relationship is unlikely attributable solely to confounding factors. Nonetheless, the studies included in this analysis exhibited variability in the extent of covariate adjustment, with one study providing only univariate results, which limits the ability to comprehensively confirm the independence of the effect across all studies. Notably, the findings indicated a more pronounced association in studies from Asian countries and those utilizing polysomnography or pulse oximetry for diagnosis, compared to those relying on administrative coding. This underscores the importance of diagnostic accuracy in risk estimation. Collectively, these results suggest that SDB should not be regarded merely as a comorbid condition in dialysis patients but rather as a clinically significant risk factor contributing to their poor prognosis.

Several pathophysiological and clinical mechanisms may elucidate the observed association. Intermittent hypoxia, a defining characteristic of SDB, activates the sympathetic nervous system, induces oxidative stress, promotes systemic inflammation, and leads to endothelial dysfunction—factors that accelerate cardiovascular disease progression [32, 33]. In patients with ESRD on dialysis, these effects may be exacerbated by uremia, anemia, chronic inflammation, and fluid overload [34]. SDB can worsen nocturnal blood pressure surges, impair left ventricular diastolic function, and heighten the risk of arrhythmias, further increasing cardiovascular risk [35]. Additionally, fluid redistribution from the lower extremities to the neck during recumbency may narrow the upper airway, intensifying obstructive events in dialysis patients [36–38]. Clinically, SDB often manifests with nonspecific symptoms such as fatigue and reduced exercise tolerance, which may be overlooked in the dialysis context, resulting in delayed diagnosis and intervention [39].

Subgroup analyses provide further insights. The stronger association observed in Asian cohorts may reflect variations in craniofacial structure, body composition, prevalence of certain comorbidities, and dialysis practices, all of which can influence both the incidence and severity of SDB [40, 41]. However, the heightened association between SDB and mortality in these cohorts necessitates careful interpretation. The Asian studies included in this meta-analysis originated from Korea, Hong Kong, Taiwan, and Japan. While these countries differ in healthcare systems and dialysis delivery, they share certain epidemiological and clinical characteristics: dialysis patients often present with lower body mass index (BMI) and higher prevalence of hypertension and diabetes compared to their Western counterparts, potentially amplifying the cardiovascular risks associated with SDB. Additionally, dialysis practices differ, with PD being more prevalent in Asia. All Asian cohorts in our analysis comprised patients with PD, whereas non-Asian studies predominantly included HD patients. This disparity in dialysis modality likely contributed to the observed subgroup differences. Moreover, the relatively long follow-up duration in the PD cohorts (41–70 months) may have heightened the likelihood of capturing adverse outcomes. Consequently, factors such as population characteristics, dialysis modality, and follow-up duration should be considered when interpreting regional subgroup findings, and caution is warranted when generalizing these results to all dialysis populations. Conversely, the more modest association in studies employing ICD coding may result from misclassification, underdiagnosis, or inclusion of milder cases [42]. The diagnostic method significantly influenced effect size, with polysomnography and oximetry revealing stronger associations than ICD coding, likely reflecting misclassification or under ascertainment in administrative data that can dilute true associations. By prioritizing objectively measured SDB in our primary analysis, our findings more accurately capture the prognostic impact of SDB in dialysis populations. Additionally, the absence of significant differences between prospective and retrospective studies, as well as between HD and PD populations suggests that the adverse impact of SDB is broadly applicable across dialysis modalities and study designs. Sensitivity analyses excluding the large Prabu study [25] substantially reduced heterogeneity, indicating that sample size and data source can influence pooled effect estimates while maintaining a consistent association direction.

This study possesses several notable strengths. It represents the most comprehensive and up-to-date synthesis of longitudinal cohort studies examining SDB and prognosis in dialysis patients. The inclusion of only studies with longitudinal follow-up strengthens the temporal relationship between SDB and subsequent adverse outcomes. Furthermore, the analysis incorporated extensive subgroup and sensitivity analyses, enhancing the confidence in the stability and generalizability of the results. The overall quality of the included studies was high, with all scoring ≥ 7 on the NOS, ensuring methodological rigor. However, certain limitations merit consideration. There was substantial heterogeneity in methods and criteria for diagnosing SDB across studies, ranging from gold-standard polysomnography to overnight pulse oximetry and administrative coding. Although diagnostic criteria varied (e.g., AHI vs ODI or SaO_2_ thresholds), all studies relied on objective measures of disordered breathing or hypoxemia. This variability precluded strict subgrouping by diagnostic index, yet the consistent associations across definitions suggest that the prognostic impact of SDB is robust. Additionally, dialysis-specific design features varied among studies; some defined the time origin as dialysis initiation, while others enrolled patients after varying periods on dialysis, thereby mixing incident and prevalent populations. This inconsistency may contribute to survivor bias and could affect hazard estimates. Due to the unavailability of individual participant-level data, we were unable to conduct sensitivity analyses focused on incident dialysis cohorts. Furthermore, while most studies adjusted for major confounders such as age, sex, comorbidities, and dialysis-related factors, residual confounding from unmeasured variables (e.g., SDB severity, treatment adherence, and socioeconomic status) is likely. Although demographic factors and major comorbidities were generally adjusted for, important variables such as dialysis adequacy (e.g., Kt/V and ultrafiltration), inflammatory status, nutritional indices, and socioeconomic factors were not consistently accounted for. These unmeasured or variably adjusted confounders could bias the observed associations, either attenuating or exaggerating the true effect of SDB on mortality and cardiovascular outcomes. This limitation emphasizes the need for future prospective studies with more comprehensive adjustment. Additionally, because all included studies were observational, causality cannot be established. Data regarding the timing of SDB diagnosis in relation to dialysis initiation, the impact of SDB treatment, and cause-specific mortality were limited, hindering more detailed mechanistic exploration. Furthermore, the definition of MACE was not fully standardized across studies, and key analytical details—such as whether only first events were counted or how kidney transplantation was managed (censoring vs competing risk)—were generally not reported. This variability limits the interpretability of the pooled secondary outcome and should be considered when applying our findings. Moreover, although we searched three major databases (PubMed, Embase, Web of Science), Scopus was not included. Given the overlap in coverage, the risk of missing eligible studies is small but cannot be entirely excluded. Finally, the limited number of studies reporting MACEs restricted the statistical power to detect heterogeneity or publication bias for this outcome. While no evidence of publication bias was detected for mortality, the modest number of included studies limits statistical power; thus, funnel plot symmetry should be interpreted cautiously. For MACEs, with only five studies, publication bias assessment remained descriptive.

From a clinical perspective, the findings underscore the importance of recognizing and addressing SDB as a critical component of comprehensive care for dialysis patients. Given its high prevalence within this population and its strong association with adverse outcomes, routine screening for SDB may facilitate earlier identification and intervention. Portable sleep monitoring and validated questionnaires could be integrated into dialysis units to enhance case finding, followed by confirmatory polysomnography when indicated. Treatment of SDB, particularly obstructive sleep apnea, with continuous positive airway pressure (CPAP) has the potential to improve patient outcomes. Notably, a recent observational study involving Japanese dialysis patients with SDB demonstrated that CPAP use was associated with nearly a 50% reduction in all-cause mortality compared with non-use, even after adjusting for age, sex, comorbidities, and AHI [43]. These findings suggest that targeted interventions may mitigate some of the excess risks associated with SDB. However, these results should be interpreted with caution, as observational studies are subject to residual confounding and cannot establish causality. Pragmatic randomized or stepped-wedge trials are necessary to determine whether systematic screening and treatment of SDB genuinely improve survival and cardiovascular outcomes in this population. Future research should also explore the optimal timing of screening (pre- vs post-dialysis initiation), the role of individualized treatment approaches, and the impact of adherence to CPAP or alternative therapies. Investigating the interplay between SDB, fluid status, cardiovascular function, and dialysis parameters could yield valuable insights into patient-specific risk modification strategies.

## Conclusion

In conclusion, this meta-analysis demonstrates that SDB is associated with increased risks of all-cause mortality and MACEs in patients receiving dialysis. While causality cannot be inferred from observational evidence, the consistency of the findings suggests that SDB may represent a potentially modifiable risk factor in this vulnerable population. Emerging observational data also indicate that treatment with CPAP could be linked to lower mortality, but randomized trials are needed to confirm this effect. These results support systematic screening for SDB in dialysis units and highlight the necessity for pragmatic interventional studies, including randomized or stepped-wedge designs, to determine whether early detection and management of SDB can improve patient outcomes.

## Supplemental data

Supplemental data are available at the following link:

https://www.bjbms.org/ojs/index.php/bjbms/article/view/13100/4020.

## Data Availability

All data generated or analyzed during this study are included in this published article.
